# Cigarette Smoke Extract Induces p38 MAPK-Initiated, Fas-Mediated Eryptosis

**DOI:** 10.3390/ijms232314730

**Published:** 2022-11-25

**Authors:** Ignazio Restivo, Alessandro Attanzio, Ilenia Concetta Giardina, Francesca Di Gaudio, Luisa Tesoriere, Mario Allegra

**Affiliations:** 1 Department of Biological, Chemical and Pharmaceutical Science and Technologies, Università degli Studi di Palermo, Via Archirafi 28, 90123 Palermo, Italy; 2 Department of Health Promotion, Mother and Child Care, Internal Medicine and Medical Specialties, Università degli Studi di Palermo, Piazza delle Cliniche 2, 90127 Palermo, Italy

**Keywords:** eryptosis, cigarette smoke, death-inducing signaling complex (DISC), p38 MAPK, ceramide, caspases

## Abstract

Eryptosis is a physiological mechanism for the clearance of senescent or damaged erythrocytes by phagocytes. Excessive eryptosis is stimulated under several pathologies and associated with endothelial injury and thrombosis. Cigarette smoke (CS) is an established risk factor for vascular diseases and cigarette smokers have high-levels of eryptotic erythrocytes. This study, for the first time, investigates the mechanism by which CS damages red blood cells (RBCs). CS extract (CSE) from commercial cigarettes was prepared and standardized for nicotine content. Cytofluorimetric analysis demonstrated that treatment of human RBCs with CSE caused dose-dependent, phosphatidylserine externalization and cell shrinkage, hallmarks of apoptotic death. CSE did not affect cellular levels of Ca^2+^, reactive oxygen species (ROS) or glutathione (GSH). Immununoprecipitation and immunoblotting revealed the assembly of the death-inducing signaling complex (DISC) and oligomerization of Fas receptor as well as cleaved caspase-8 and caspase-3 within 6 h from the treatment. At the same time-interval, CSE elicited neutral sphyngomielinase (nSMase) activity-dependent ceramide formation and phosphorylation of p38 MAPK. Through specific inhibitors’ nSMase, caspase-8 or p38 MAPK activities, we demonstrated that p38 MAPK activation is required for caspase-8-mediated eryptosis and that ceramide generation is initiator caspase-dependent. Finally, ex vivo analysis detected phosphorylated p38 MAPK (p-p38) and Fas-associated signaling complex in erythrocytes from cigarette smokers. In conclusion, our study demonstrates that CSE exposure induces in erythrocytes an extrinsic apoptotic pathway involving p38 MAPK-initiated DISC formation followed by activation of caspase-8/caspase-3 via ceramide formation.

## 1. Introduction

Mature RBCs undergo a controlled suicidal death program, also known as eryptosis, which is in many aspects comparable to apoptosis of nucleated cells. The distinctive features of eryptotic cells include cell shrinkage, membrane blebbing, activation of proteases and membrane lipid scrambling leading to phosphatidylserine (PS) externalization to the outer membrane leaflet [1].

Eryptosis is a physiological process, essential for the clearance of senescent or damaged circulating erythrocytes by phagocytes. However, a dysfunctional and excessive RBC’s apoptosis is associated to vaso-occlusive complications and, presumably, participates in the development of those vascular disfunctions that are associated with heart failure [2,3], chronic kidney disease [4,5] and metabolic syndrome [6]. Indeed, PS at the surface of eryptotic erythrocytes favours their adherence to endothelial cells as well as platelets by transmembrane CXCL16 scavenger receptor, potentially impeding the microcirculation as well as triggering blood clotting and thrombosis [7,8,9]. In recent years, there has been a continuous and rapid growth in the field of eryptosis-related studies aimed at ascertaining the conditions that stimulate the cellular process and the underlying mechanisms.

Eryptosis is triggered by complement, hyperosmotic shock, energy depletion, redox unbalance, increase of temperature and by a wide variety of xenobiotics [10,11,12]. Increases of cytosolic Ca^2+^ activity, prostaglandin E_2_ (PGE_2_)-activated cation channels, ceramide, caspase-3, calpain, membrane receptors for glutamate or leukotrienes and a number of protein kinases (e.g., AMPK, JAK3, PKC, p38 MAPK) have been established as endogenous mediators of eryptosis [3]. Interestingly, although RBCs express the protein components of the DISC such as death membrane receptors, exemplified by Fas, death-linked adapter proteins (FADD) and caspase-8 [13], the Fas-mediated death cascade of mature RBCs still remains controversial, with evidence for or against [14,15].

CS is a well-established risk factor in the aetiology of cardiovascular diseases, including peripheral vascular disease [16], ischemic heart disease [17], atherosclerosis [18], myocardial infarction [18] and stroke [19]. Knowledge of the molecular mechanisms responsible for the CS-induced vascular dysfunction is still limited, although it is known that tobacco smoke components cause damage to endothelial cells, leukocytes and platelets [20,21,22,23].

In a recent study, we have reported that cigarette smokers have high levels of circulating eryptotic erythrocytes, which suggests a possible contribution of eryptosis to the smoke-associated vascular injury [24]. To assess a causative association linking CS to eryptosis, in this in vitro study, we investigated whether CSE may directly affect the RBCs life and determined the mechanism by which this occurs. We show that CSE triggers eryptosis through an extracellular signaling pathway, with DISC assembly leading to the nSMase-dependent ceramide release and caspase-8/caspase-3 activation. This cascade of events appears to be initiated by p38 MAPK. These results were corroborated by ex vivo evidence that RBCs isolated from smoking subjects show p-p38 MAPK and Fas-associated signaling complex.

## 2. Results

### 2.1. CSE Induces Oxidative Stress-Independent Eryptosis

**Figure 1:**
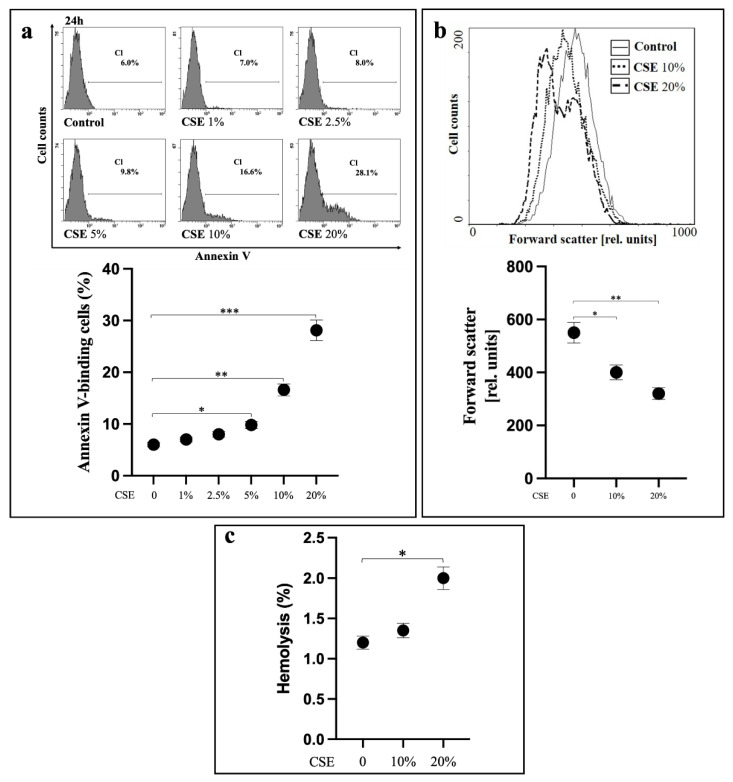
CSE induces eryptosis. (**a**) Percentage of PS-exposing erythrocytes, (**b**) their FSC and (**c**) percentage of cell haemolysis after 24 h incubation with CSE. RBCs incubated with vehicle were used as control. Values are means ± SD of six independent experiments carried out in triplicate. * *p* < 0.05; ** *p* < 0.01; *** *p* < 0.001 (ANOVA associated with Tukey’s test).

Loss of membrane phospholipid asymmetry in erythrocytes leading to PS externalization is a hallmark of eryptosis. When human RBCs were treated with CSE from 0 to 20% for 24 h, a dose-dependent induction of PS externalization, evaluated by annexin V- fluorescein isothiocyanate (FITC) staining, was evident (Figure 1a). After a 24 h exposure with either 10 or 20% CSE, annexin V binding to RBCs accounted for 17.1 ± 0.91% or 27.6 ± 1.55%, respectively. In these cells, a net decrease in volume, determined by forward scatter (FSC), was also observed (Figure 1b). Haemoglobin (Hb) release from RBCs treated with either 10 or 20% CSE was measured as an index of membrane fragility associated to cell necrosis. With respect to untreated cells (control), CSE treatment induced a slight but significant release of Hb only at 20%, with a value increasing from 1.2% ± 0.1% to 2.0 ± 0.3% (Figure 1c).

**Figure 2:**
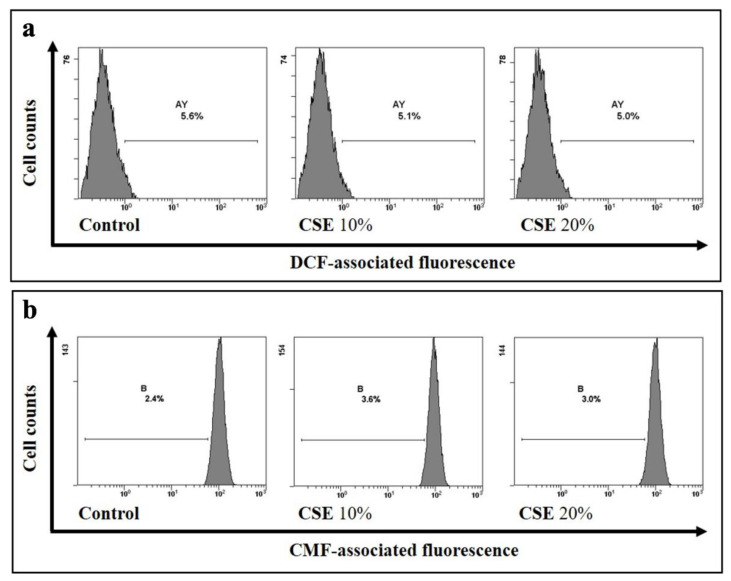
CSE does not induce oxidative stress in erythrocytes. (**a**) ROS levels and (**b**) GSH content in RBCs after 24 h incubation with CSE. RBCs incubated with vehicle were used as control. Representative images of four experiments carried out in triplicate with comparable results.

CSE contains more than 5000 oxidizing chemicals as well as a very high number of unidentified long- or short-lived free radicals [25]. We then measured ROS and GSH levels in RBCs exposed to CSE. Cytofluorimetric measurements using specific probes showed that, in comparison to control cells, incubation for 24 h with either 10 or 20% CSE induced no significant variation (*p* > 0.05, n = 6) of the intracellular levels of ROS neither caused GSH depletion (Figure 2).

### 2.2. CSE Induces Eryptosis through Extrinsic Apoptotic Pathway

**Figure 3:**
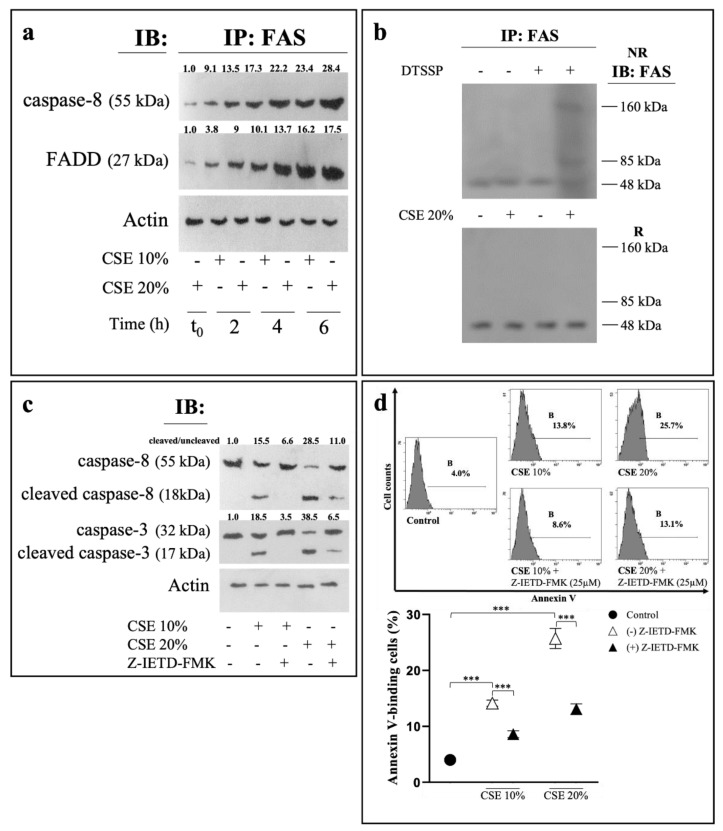
CSE induces eryptosis through extrinsic signaling pathway activation. (**a**) RBCs treated at different time intervals with CSE were lysed and immunoprecipitated with anti-Fas antibody. The immunoprecipitates (IP) were separated by SDS-PAGE and blotted with anti-FADD or anti-caspase-8. Blots were reprobed with anti-Fas antibody to ensure equal loading of the gels. (**b**) Oligomerization of Fas. Thirty min before the end of treatment (6 h) with CSE, RBCs were either left untreated or treated with the thiol-cleavable cross-linker DTSSP (1 mM). The IP with anti-Fas were separated by SDS-PAGE under NR and R conditions and blotted with anti-Fas antibody. Blots are representative of the results obtained in three separate experiments with comparable results. (**c**) Western blot analysis of caspases-8/3. The numbers below each line represent the relative expression level normalised to actin, as determined on the basis of the intensity of the band. Representative image of three experiments with similar results. (**d**) Percentage of PS-exposing cells in RBCs pre-incubated for 1 h in the absence or in the presence of the caspase-8 inhibitor Z-IETD-FMK (25 μM) before the treatment with CSE for 6 or 24 h, respectively. RBCs incubated with vehicle were used as control. Values are means ± SD of six independent experiments carried out in triplicate. *** *p* < 0.001 (ANOVA associated with Tukey’s test).

In order to evaluate formation of DISC in membranes, RBCs treated with either 10 or 20% CSE were lysated and then immunoprecipitated with anti-Fas antibody, followed by Western blotting with either anti-FADD or anti-caspase-8 antibody. Both caspase-8 and FADD were found in the immunoprecipitated from RBCs treated with CSE, the longer the exposure time or the greater the CSE amount, the higher the protein amounts (Figure 3a).

Because Fas receptor aggregation is associated with the recruitment of DISC, receptor oligomerization in RBCs was evaluated after a 6 h treatment by adding (3,3′-Dithiobis (sulfosuccinimidyl propionate)) (DTSSP). Cell lysates were then immunoprecipitated with anti-Fas antibody and separated by SDS-PAGE under nonreducing (NR) and reducing (R) conditions. As shown in Figure 3b, immunoblotting with anti-Fas antibody of the separated proteins revealed bands at high molecular weight (80–150 kDa) in NR gels in CSE-treated RBCs but not in control erythrocytes.

Caspase-8 transduces apoptotic signals originating from DISC by undergoing autocleavage and processing the executioner caspase-3 [26]. After exposing RBCs to either 10 or 20% CSE for 6 h, cell lysates were immunoblotted with anti-caspase-8 or caspase-3. In comparison with control cells, CSE caused a decrease of the levels of the inactive precursors of both caspases, with the concomitant appearance of the cleaved active caspase-8 p18 subunit and the cleaved caspase-3 p17 subunit (Figure 3c). Pre-incubation of RBCs with the caspase-8 specific inhibitor Z-IETD-FMK before exposure to CSE, prevented the formation of the active caspase-3 subunit (Figure 3c). Moreover, Z-IETD-FMK significantly reduced the annexin V-FITC associated fluorescence measured after 24 h treatment (Figure 3d) in comparison with RBCs pre-incubated in the absence of the inhibitor. Interestingly, Z-IETD-FMK did not modify the release of Hb caused by the CSE treatment (not shown), excluding that necroptosis, normally antagonized by caspase-8 [27], was involved in the CSE-induced death of RBCs.

### 2.3. CSE-Induced Eryptosis Requires Caspase-8-Mediated Ceramide Formation

**Figure 4:**
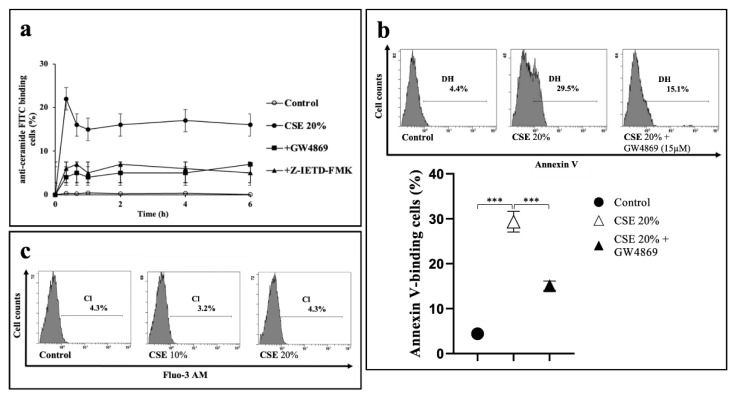
CSE-induced eryptosis requires caspase-8-mediated ceramide formation and is Ca^2+^ independent. (**a**) Time-course of ceramide formation in 20% CSE-treated RBCs pre-incubated for 1 h in the absence or in the presence of nSMase inhibitor GW4869 (15 μM) or caspase-8 inhibitor Z-IETD-FMK (25 μM). (**b**) Percentage of PS-exposing RBCs after 1 h pre-treatment in the absence or in the presence of GW4869 (15 μM) followed by 24 h treatment with CSE. Values are means ± SD of six independent experiments carried out in triplicate. *** *p* < 0.001 (ANOVA associated with Tukey’s test). (**c**) Analysis of intracellular calcium levels. Representative of four experiments carried out in triplicate with comparable results. RBCs incubated with vehicle were used as control.

A sphingolipid-imbalance in membrane leading to ceramide release is a condition associated to eryptosis [28]. We evaluated the time-course of ceramide formation in RBCs exposed to 20% CSE in a 0–6 h time interval by FACS analysis. Compared to control cells, exposure of RBCs to CSE caused a very rapid increase of ceramide levels that peaked at 20 min of treatment (Figure 4a).

A number of factors related to morphological changes and apoptosis may elicit nSMase activity in human RBCs [29]. When RBCs were pre-incubated for 1 h with the nSMase inhibitor GW4869 before the stimulation with CSE, ceramide synthesis was strongly inhibited (Figure 4a), while a net decrement of PS externalized, expressed as % of annexin V-binding cells, was also measured after 24 h (Figure 4b). Interestingly, also pre-treatment of the cells with the caspase-8 inhibitor Z-IETD-FMK resulted in a reduction of ceramide formation (Figure 4a). While indicating the involvement of nSMase in the eryptotic process, our findings also point out that in the CSE-induced signaling pathway, ceramide generation appears to be dependent by the initiator caspase.

It has been reported that ceramide sensitizes erythrocytes to the eryptotic activity of calcium ions [28]. As shown in Figure 2c, a 24 h treatment of RBCs with 20% CSE did not cause a significant increase of the cytosolic calcium level. We also exposed the cells to CSE in the absence of extracellular Ca^2+^ and in the presence of the cell permeable calcium-chelating agent BAPTA-AM (50 μM). Under this condition, the PS exposure was comparable with that of the CSE-treated cells in a medium containing Ca^2+^.

### 2.4. CSE-Induced Extrinsic Eryptosis Is Initiated via p38 MAPK

**Figure 5:**
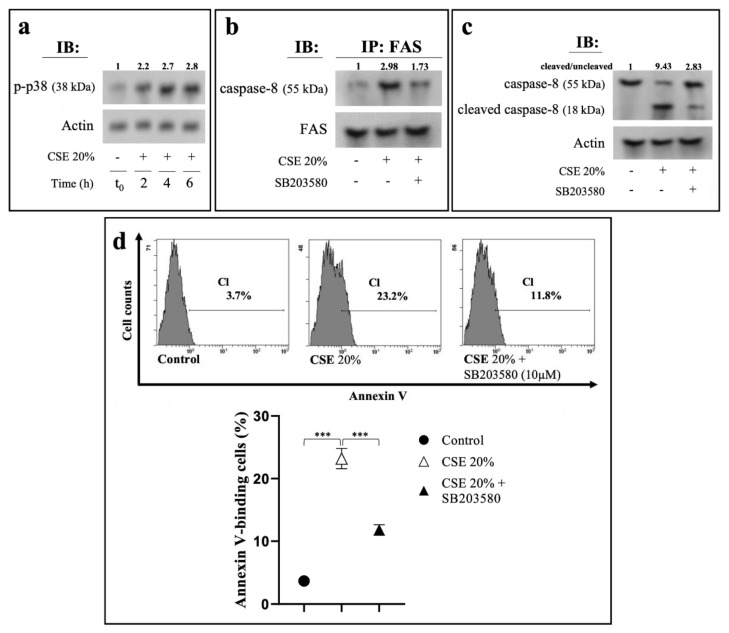
CSE-induced eryptosis is dependent by p38 MAPK activation. (**a**) Western blot analysis of p-p38 in RBC treated at different time intervals with CSE. (**b**) Association of Fas with caspase-8 in RBC treated with CSE in the absence or in the presence of p-38 MAPK inhibitor. RBCs were pre-incubated for 1 h in the absence or in the presence of 10 μM SB203580 before 6 h treatment with 20% CSE. Immunoprecipitation with anti-Fas antibody and immunodetection of caspase-8. Blots were reprobed with anti-Fas antibody to ensure equal loading of the gels. (**c**) Western blot analysis of caspases-8. The numbers below each line represent the relative expression level normalized to actin, as determined on the basis of the intensity of the band. Representative image of three experiments with similar results. (**d**) Percentage of PS-exposing cells in RBCs pre-incubated for 1 h in the absence or in the presence of SB203580 (10 μM) before the treatment with CSE for 6 or 24 h, respectively. RBCs incubated with vehicle were used as control. Values are means ± SD of six independent experiments carried out in triplicate. *** *p* < 0.001 (ANOVA associated with Tukey’s test).

Activation of p38 MAPK is associated with eryptosis induced by different cell stressors [30]. CSE treatment caused an evident increase of the active p-p38 from 0 to 6 h of exposure (Figure 5a). To investigate the functional involvement of p38 MAPK in the CSE-induced eryptotic mechanism, we pre-incubated RBCs with SB203580, a specific inhibitor of the enzyme. Inhibition of p38 kinase significantly decreased the amount of procaspase-8 in the immunoprecipitated with anti-Fas (Figure 5b) as well as of its cleaved active p18 subunit in the cell cytosol (Figure 5c). In addition, in comparison with CSE-stimulated RBCs in the absence of SB203580, we measured a reduction of the annexinV-FITC associated fluorescence (Figure 5d).

### 2.5. Ex Vivo Analysis of Erythrocytes from Smokers

**Figure 6:**
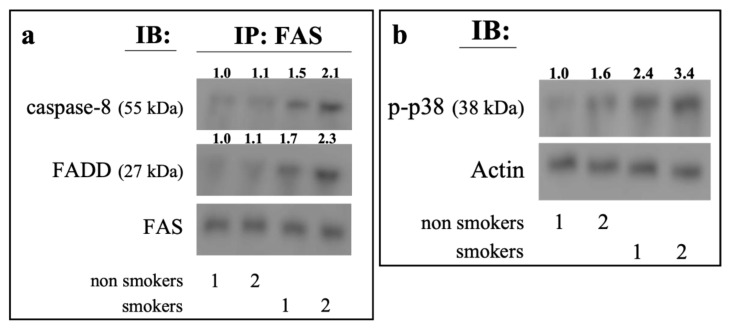
Fas-associated signaling complex and p-p38 MAPK levels in erythrocytes from smokers and healthy humans. RBCs isolated both from smokers and non-smokers volunteers were lysed and submitted either to immunoprecipitation with anti-Fas antibody (**a**) or to Western blot analysis of p-p38 MAPK (**b**). The IP were separated by SDS-PAGE and blotted with anti-FADD or anti-caspase-8. Blots were reprobed with anti-Fas (**a**) or anti-actin (**b**) antibody to ensure equal loading of the gels. The numbers below each line represent the relative expression level normalised to actin, as determined on the basis of the intensity of the band. Representative image of three experiments with similar results.

Smokers have high levels of eryptotic circulating erythrocytes [24]. To corroborate the role of the CSE-induced, Fas-dependent eryptotic pathway, we looked for a DISC-like complex in erythrocytes from smokers. With this aim, isolated RBCs from healthy smoking or non-smoking volunteers were submitted to co-immunoprecipitation experiments with anti-Fas followed by immunoblotting for FADD and caspase-8. Densitometric analysis showed a significantly higher localization of both the proteins in the IP of RBCs isolated from smokers with respect to those from non-smokers (Figure 6a). Moreover, erythrocytes from smokers showed higher levels of p-p38 MAPK than those from non-smokers, (Figure 6b).

## 3. Discussion

Numerous studies link cigarette smoke to cardiovascular system injury that leads to pathological changes including endothelial cell dysfunction, leucocyte and platelet activation as well as increase of circulating eryptotic RBCs [20,21,23,24]. Despite many efforts have been done to understand how CS causes direct cytotoxicity, the exact mechanisms remain yet unclear and often controversial. In the current study we demonstrate that CSE exposure induces in erythrocytes an extrinsic apoptotic pathway involving p38 MAPK-initiated DISC formation followed by activation of caspase-8/caspase-3 via ceramide formation. Male blood volunteers have been selected to avoid hormonal changes related to the ovarian and menstrual cycle that can play a critical role in RBC signaling as previously reported [31]. To the best of our knowledge, this is the first study investigating the mechanism by which CS can affect erythrocytes health and fate.

CSE, simulating the various harmful components of real CS, includes high concentrations of oxidants and ROS inducers [25]. Notwithstanding oxidative stress has been recognized as key mechanism of CSE toxicity in various human cells, including alveolar macrophages [32], bronchial epithelial cells [33] and lung fibroblasts [34], we here show that CSE causes cell shrinkage and PS externalization in RBCs without affecting the ROS/GSH balance. In line with our data, other studies in endothelial cells have demonstrated that CSE treatment has no effect on GSH content [35] and that antioxidants exhibit only a minimal protective effect in CSE-triggered cell death [36]. In addition, our data exclude that activities of enzymes that generate reactive either oxygen or nitrogen species in the erythrocytes, such as NADPH oxidase, nitric oxide synthase or xanthine oxido-reductase, are up-regulated by the CSE-treatment [37]. However, RBC isolated from smokers’ subjects showed reduced GSH levels with respect to those in non-smokers ones [24]. Possibly, eryptosis in smokers’ subjects depends on multifactorial and systemic factors other than a direct toxicity of smoke components.

Mitochondrial dysfunction resulting in the release of apoptogenic factors has been recognized as the main apoptotic death signaling mediated by CSE. However, molecular investigations have demonstrated that CSE can also sensitize cells toward apoptosis via elements of the extrinsic pathway [38]. While lacking mitochondria, mature erythrocytes contain all the components of the receptor-dependent apoptotic pathway [14]. Though it has been ascertained that the Fas/FasL pathway plays an important role in the inhibition of the expansion and differentiation of immature erythroblasts [39,40], functional role of the extrinsic apoptotic pathway in mature erythrocytes is still debated. Indeed, Fas receptor ligation with agonistic antibody was shown to have no impact on either caspase-8 activation [13] or PS exposure [15]. Conversely, Mandal and coll. [14] have clearly demonstrated that oxidative stress leads to the formation of Fas-FADD-caspase-8 complexes in RBCs membrane, followed by caspase-8/3 activation and PS externalization to the outer membrane leaflet. In addition, the activation of ROS-associated Fas signaling pathway has been demonstrated in erythrocytes from animals chronically exposed to arsenic or lead [41,42]. Within this controversial scenario, in the present study we demonstrate that CSE induces Fas aggregation and that the FADD and caspase-8 co-immuneprecipitate with Fas in RBCs, consistently with formation of DISC. Moreover, the activity of initiator caspase-8 appears a prerequisite for both caspase-3 activation and PS externalization, supporting the involvement of a Fas/caspase-8/caspase-3-dependent signaling in CSE-induced eryptosis. As a whole, our data demonstrate for the first time that the Fas-mediated signaling cascade in mature erythrocytes can be an integral and functional mechanism of death pathway not mediated by oxidative stress.

Previous studies have provided evidence that ceramide generation is critically involved in CSE-induced cytotoxicity in numerous human cultured cells as well as in vivo. nSMase activation appears required for the CS-mediated upregulation of ceramide levels and apoptosis in human airway epithelial cells [43], rat microvascular cells [44] and in lung tissues from smokers with emphysema [45]. Furthermore, nSMase activity and ceramide formation in erythrocytes has been involved in the mechanism of Pb(II) toxicity [46]. In line with this evidence, our study shows that nSMase-dependent formation of ceramide occurs in CSE-treated RBCs and that this lipid mediator is a key player in the eryptotic mechanism. Indeed, nSMase inhibition by GW4869 reduced both ceramide formation and PS externalization. Ceramide is considered an indispensable apoptotic signal in the Fas signaling pathway, demonstrated by the tendency of signaling receptors to cluster in ceramide-enriched platforms [47]. The role of ceramide in the CSE-induced Fas aggregation in RBCs is actually under investigation in our laboratory. In addition, our data indicate that ceramide generation depends on caspase-8 action, according to consolidated evidence indicating that, at early stage of Fas signaling, this initiator protease is essential to activate sphingomyelinase [48,49,50].

nSMase-dependent ceramide generation, linked to increased intracellular calcium levels and scramblase activation, has been reported as a key mechanism underlying heavy metal-induced eryptosis [46]. Interestingly, in our study eryptotic activity of CSE does not involve calcium entry and, consequently, scramblase activity can be excluded from the breakdown of PS asymmetry.

p38 MAPK is a prominent mediator of the cellular stress response and is considered an important element of the cellular machinery triggering the apoptosis of nucleated cells [51] as well as of erythrocytes [30]. It has been reported that exposure to CS induces phosphorylation of p38 followed by activation of Fas-dependent signaling pathway and then apoptosis in lung tissue of rats [52]. In erythroblasts, the ASK1/JNK/p38 signaling axis is required for the Fas-mediated caspase activation that drives erythroid differentiation [53].

**Figure 7:**
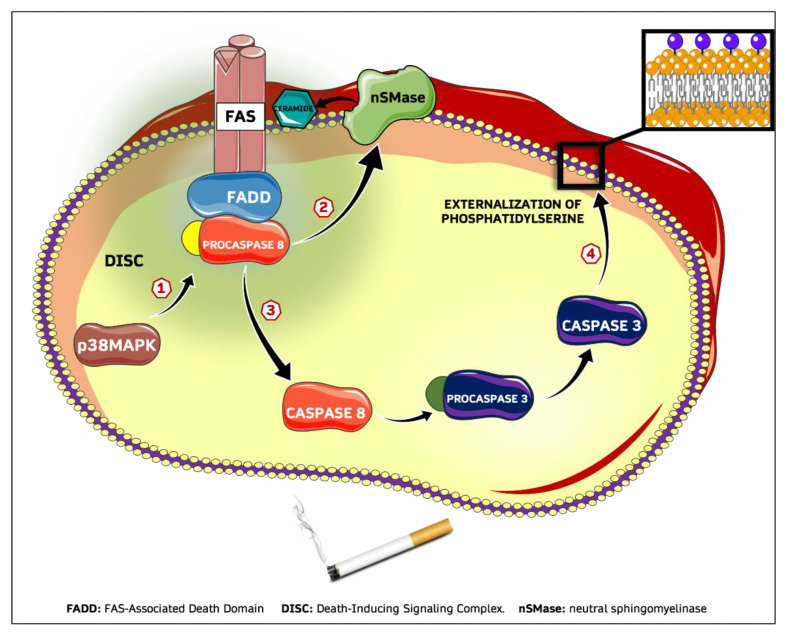
Proposed mechanism for CSE-induced eryptosis. **1**: Phosphorylation of p38 MAPK activates DISC assembly with activation of caspase-8; **2**: Ceramide production by nSMase is regulated by procaspase-8; **3**: Trimerization of Fas and binding with FADD and procaspase-8 is followed by the caspase activation cascade; **4**: Active subunit of caspase-3 causes loss of asymmetry of membrane phospholipids with final phosphatidylserine externalization.

Although many aspects of the CSE-induced eryptotic mechanism still need to be clarified, our data allow us to picture the possible sequence of the molecular events as represented in Figure 7.

At last, in this work we show higher levels of Fas-associated FADD and caspase-8, as well as of p-p38MAPK levels, in erythrocytes from two smoker subjects in comparison with those from not smokers, supporting the Fas-dependent signaling pathway in CS-dependent eryptosis. Analysis on a larger sample of subjects is, however, necessary to unequivocally validate this finding.

In conclusion, our study provides evidence that, in mature erythrocytes, stimulation of p38 MAPK by CSE may sensitizes the cells to Fas-induced caspase activation driving the cell fate toward programmed death.

### Study Limitations

While a major aspect of the present study is to investigate for the first time the mechanism by which CS can affect RBC health and fate, some limitations should be considered. Firstly, while our data suggest that scramblase activity can be excluded from the breakdown of PS asymmetry, involvement of the aminophospholipid translocase activity in the CSE-induced eryptotic mechanism deserves to be investigated. Secondly, in the present study, we show that CSE-induced eryptosis in RBCs is initiated by activation of p38 MAP kinase leading to DISC assembly and caspase cascade. The possible role of ASK1 as upstream molecule capable of regulating p38 MAPK remains to be investigated. At last, the ex vivo experiments supports the hypothesis that CS may induce the formation of complexes involving death receptors and death-linked adapter proteins in RBC. The sample size supporting this data is, however, small. Nevertheless, we believe this result may pave the way for further investigations, some of which are actually in progress in our lab.

## 4. Materials and Methods

When not specified, all chemicals were purchased from Sigma-Aldrich (Milan, Italy) and were of the highest purity grade available.

### 4.1. Preparation of CSE

CSE was prepared by a modification of a previously published method [35]. Briefly, 3 filtered commercial Marlboro Red cigarettes (Philip Morris USA Inc., Richmond, VA, USA), each containing 0.8 mg nicotine, 10 mg tar and 10 mg carbon monoxide according to the manufacturer’s report, were smoked consecutively through an apparatus with a constant airflow (0.4 L/min) controlled by an air compressor. The smoke was bubbled through 30 mL of Ringer solution warmed to 37 °C. The Ringer solution contained (mM) 125 NaCl, 5 KCl, 1 MgSO_4_, 32 N-2-hydroxyethylpiperazine-N-2-ethanesulfonic acid (HEPES)/NaOH, 5 glucose, 1 CaCl_2_, pH 7.4. Cigarettes were combusted up to 3 mm from the filter. The smoke solution was adjusted to pH 7.4 and then filtered through a 0.22-mm filter (Millipore, Bedford, MA, USA). This solution was considered to be 100% CSE and used within 30 days. Concentration of nicotine, one of the stable constituents, was assessed by LC–MSMS analysis carried out using an Ultimate 3000 UHPLC system coupled to a TSQ Quantiva triple quadrupole mass spectrometer (Thermo Fisher Scientific, San José, CA, USA). Nicotine mean concentration of three different CSE preparations was 32.13 ± 1.23 mg/mL.

### 4.2. RBCs and Treatment

Fresh blood samples were collected in heparinized tubes from non-smoking healthy male volunteers (n = 8; age range 33–66 years; normal BMI range) with informed consent, and RBCs were immediately collected by centrifugation (2000× *g*, 4 °C, 20 min) over a Ficoll (Sigma-Aldrich, Cat. No. F5415) gradient. Cells were washed twice in Ringer solution. Aliquots of the cell pellet were diluted to 0.4% haematocrit (HT) in the same buffer (control) or in Ringer containing CSE at appropriate concentration for the treatment. Erythrocytes were then incubated at 37 °C, 5% CO_2_ and 95% humidity for the indicated times. For treatments in nominally calcium-free Ringer, CaCl_2_ in the solution was replaced by 50 μM cell-permeable calcium-chelating agent BAPTA-AM (Sigma-Aldrich, Cat. No. A1076).

Where indicated, pre-treatment was carried out with either nSMase inhibitor GW4869 (Sigma-Aldrich, Cat. No. D1692) (15 μM), caspase-8 inhibitor Z-IETD-FMK (MedChemExpress, Monmouth Junction, NJ, USA, Cat. No. HY-101297) (25 μM) or p38 MAPK inhibitor SB203580 (MedChemExpress, Cat. No. HY-10256) (10 μM). Inhibitors were added in dimethyl sulfoxide (DMSO) at a 0.1% (v:v) final concentration at the RBCs samples (HT 0.6%). Cells were pre-incubated for 1 h at 37 °C before that CSE at appropriate amount, or Ringer (control), was added. HT was then corrected at 0.4% and the treatment was prolonged until the indicated times.

The experimental study protocol was approved by the Ethic Committee of Palermo 1, University Hospital (No. 8-09/2022) and performed in accordance with the Declaration of Helsinki and its amendments.

### 4.3. Haemolysis

After treatment, RBCs were centrifuged (1800 rpm, 24 °C, 5 min) and Hb concentration in the supernatant was measured by absorbance at 421 nm (Soret’s band) by spectrophotometry (DU-640 Spectrophotometer Beckman, Brea, CA, USA). The absorbance value at 421 nm of the supernatant derived from similar erythrocytes lysed in distilled water was considered as 100% haemolysis.

### 4.4. Flow Cytometry

#### 4.4.1. Measurement of PS Externalization and FSC

RBCs were washed once in Ringer solution, pH 7.4, and adjusted to 1.0 × 10^6^ cells/mL with binding buffer following the manufacturer’s instructions. In experiments designed to evaluate the percentage of PS externalization, suspension of RBCs (100 µL) was incubated with 5 µL of annexin V-FITC (eBioscience, San Diego, CA, USA, Cat No. 88-8005-74) at room temperature in the dark for 15 min. Subsequently, suspension samples of at least 1.0 × 10^4^ cells were subjected to FACS analysis by Epics XL™ flow cytometer, using Expo32 software (Beckman Coulter, Fullerton, CA, USA). RBCs were analysed by FSC, and annexin V-fluorescence intensity was measured in fluorescence channel FL-1 with an excitation wavelength of 488 nm and an emission wavelength of 530 nm.

#### 4.4.2. Measurement of Intracellular ROS

Production of ROS was determined by measuring percentage changes in fluorescence resulting from oxidation of the dichloro-dihydro-fluorescein diacetate (DCFDA) (Sigma-Aldrich, Cat. No. D6883) fluorescent probe. DCFDA, at 10 µM final concentration, was added to 1.0 × 10^5^ cells 30 min before the end of the treatment, in the dark. RBCs were collected by centrifugation (2000× *g*, 4 °C, 5 min), washed, resuspended in phosphate-buffered saline (PBS), and analysed as reported by Attanzio et al. [37].

#### 4.4.3. Measurement of Intracellular GSH

Measurement of intracellular GSH level was monitored by probe 5-chloromethylfluorescein diacetate (CMFDA) (Abcam, Cambridge, UK, Cat. No. ab145459). CMFDA, at 1 µM final concentration, was added to 1.0 × 10^5^ cells 40 min before the end of the treatment, in the dark. RBCs were collected by centrifugation (2000× *g*, 4 °C, 5 min), washed, resuspended in PBS, and analysed by FACS [54].

#### 4.4.4. Measurement of Intracellular Calcium

Cytosolic concentration of Ca^2+^ was monitored by measuring the fluorescence change of the Fluo-3 AM (Sigma-Aldrich, Cat. No. 73881) dye used as a probe for Ca^2+,^ whose intensity is directly representative of the cytosolic levels of the ion. Briefly, Fluo-3 AM at 2 µM final concentration was added to 1.0 × 10^5^ erythrocytes 40 min before the end of treatment in the dark. After centrifugation, (2000× *g*, 4 °C, 5 min) RBCs were washed, resuspended in PBS and analysed as reported in Section 4.4.3.

#### 4.4.5. Measurement of Ceramide

Abundance of ceramide levels was measured as follows. Briefly, after treatment, 1.0 × 10^5^ erythrocytes were incubated for 1 h at 37 °C with 1 µg/mL of a mouse monoclonal anti-ceramide antibody (Sigma-Aldrich, Cat. No. C8104) in PBS containing 0.1% bovine serum albumin (BSA). RBCs after two washing steps with PBS-BSA, were stained for 30 min with 20 µL of a goat anti-mouse, polyclonal, FITC, secondary antibody (Millipore, Billerica, MA, USA, Cat. No. AQ502F) diluted 1/50 in PBS-BSA in the dark. Finally, erythrocytes were collected by centrifugation (2000× *g*, 4 °C, 5 min), washed twice, resuspended in PBS and analysed by flow cytometer as reported in Section 4.4.3.

### 4.5. Immunoprecipitation

RBCs (2.0 × 10^8^ cells) were washed twice with PBS, resuspended in lysis buffer (20 mM Tris-HCl, pH 7.6, 100 mM NaCl, 10 mM MgCl_2_, 1% NP-40, 2 mM PMSF, 0.5 mM DTT and 2 mg/mL lysozyme) containing phosphatase (Roche, Basel, Switzerland, Cat, No. 4906845001)- and protease inhibitors (Roche, Cat. No. 4693132001) and sonicated (2 cycles, each for 30 s) with Labsonic LBS1-10 (Labsonic Falc, Treviglio, Italy). Lysates were centrifuged (40,000× *g*, 4 °C, 1 h) and supernatants were immunoprecipitated overnight with mouse anti-Fas antibody (1:200) at 4 °C. Supernatants were then incubated with 20 μL of Protein G PLUS-Agarose (Santa Cruz, Biotechnology, Dallas, TX, USA, sc-2002) for 3 h at 4 °C. Beads were pelleted, washed twice in lysis buffer and finally proteins were separated by SDS-PAGE for immunorecognition by western blotting.

### 4.6. Western Blotting

Erythrocytes (2.0 × 10^8^ cells) were lysed, centrifuged as reported in Section 4.2 and supernatants were collected. Bradford protein assay (Bio-Rad, Hercules, CA, USA, Cat. No. 5000006) was used to quantify the total protein concentration. For each sample, equal amounts of proteins were loaded (50 µg/lane), separated on 10% gel by discontinuous SDS-PAGE and then electrotransferred to a polyvinylidene difluoride (PVDF) membrane (Millipore, Cat. No. IPVH00010). Blots were treated with blocking solution (5% non-fat dry milk) and then incubated overnight at 4 °C with primary antibodies in Tris-buffered saline (TBS; 25 mM Tris, 150 mM NaCl, pH 7.4) containing Tween 20 (1%, *v*/*v*) (TBST) and 5% (*w*/*v*) BSA. Mouse monoclonal anti-FADD (Santa Cruz, sc-271748), anti-Fas (Santa Cruz, sc-74540), anti-caspase-3 (Santa Cruz, sc-56053), anti-caspase-8 (Santa Cruz, sc-81657) and anti-p-p38 MAPK (Santa Cruz, sc-166182) primary antibodies were used at a dilution of 1:200. After washing three times with TBST, immunoblots were incubated with a 1:2000 dilution of rabbit anti-mouse IgG Antibody, horseradish peroxidase conjugated (Sigma-Aldrich, Cat. No. AP160P) for 1 h at room temperature. Immunoblots were then washed five times with TBST and developed by enhanced chemiluminescence (Amersham, Milan, Italy, Cat. No. RPN2232). Mouse monoclonal anti-actin antibody (Santa Cruz, sc-8432) was used as loading control. Densitometric analysis of protein spots was measured by Quantity One Imaging Software (Bio-Rad, Cat. No. 1708265) and the results were reported as arbitrary densitometric units normalized to actin.

### 4.7. Cross-Linking Reaction by (3,3′-Dithiobis (Sulfosuccinimidyl Propionate)) DTSSP

DTSSP (Sigma-Aldrich, Cat. No. 803200) is a synthetic impermeable thiol-cleavable cross-linker that reacts with primary amines to form stable amide bound. For cross-linking, 1 mM DTSSP in PBS was added to RBCs samples 30 min before end of the treatment. Erythrocytes were then lysed in 1% Triton X-100, 5 mM iodoacetate, protease inhibitors, 150 mM NaCl, 15 mM EDTA and 10 mM Tris-HCl as reported by Mandal et al. [13]. After immunoprecipitation with anti-Fas antibody as described in Section 2.2, samples were separated into two aliquots and boiled independently in SDS gel denaturing sample buffer, as reducing condition, and β-mercaptoethanol-free sample buffer, as non-reducing condition to distinguish between possible aspecific, denatured Fas aggregates and the functional ones. Finally, samples were separated by 7.5% SDS-PAGE and analysed with anti-Fas antibody.

### 4.8. Ex Vivo Analysis

Fresh blood samples from 2 healthy non-smoking males aged 39 and 56, and 2 males of matching age who regularly smoked 15 or more cigarettes per day for 10 years were drawn, with informed consent. Subjects had normal range of BMI and they did not report any kind of disease or drug use. Plasma cotinine level, a highly sensitive and specific biomarker of tobacco smoke exposure, was analysed using an Ultimate 3000 UHPLC system coupled to a TSQ Quantiva triple quadrupole mass spectrometer with a detection limit of 0.015 ng/mL. It accounted for 440.19 ± 18.30 ng/mL in the smoker subject number 1 and 611.83 ± 23.11 ng/mL in the smoker subject number 2 (analysis in duplicate), while it was under the detection limit in both the non-smokers volunteers. RBCs from the subjects were isolated as reported in Section 2.2 and immediately used for immunoprecipitation and Western blotting analysis.

### 4.9. Statistical Analysis

Results are expressed as mean ± SD of n separate experiments in triplicates. Statistical comparisons were performed by one-way analysis of variance (ANOVA) followed by Tukey’s correction for multiple comparisons using Prism version 8 (GraphPad Software Inc., San Diego, CA, USA). In all cases, significance was accepted if the null hypothesis was rejected at the *p* < 0.05 level.

## Data Availability

Data supporting reported results can be obtained by the corresponding author.
